# Books and Magazines

**Published:** 1910-05

**Authors:** 


### Books and Magazines.

THE PROPAGANDA FOR REFORM IN PROPRIETARY MEDICINES. Sixth edition. Containing the various exposes of nostrums and quackery which have appeared in the *Journal of the American Association.* Price, paper, 10 cents; cloth, 35 cents. Pp. 292; illustrated.

This book presents in convenient form most of the exposures that have appeared in the *Journal of the American Medical Association* showing the composition of various proprietary preparations.

---

THE SEXUAL LIFE OF WOMAN IN ITS PHYSIOLOGICAL, PATHOLOGICAL AND HYGIENIC ASPECTS. By E. Heinrich Kisch, M. D., Professor of the German Medical Faculty of the University of Prague; Physician to the Hospital and Spa of Marienbad; Member of the Board of Health, Etc. Only authorized translation into the English language from the German. By M. Eden Paul, M. D. With 97 illustrations in the text. New York: Rebman & Co. 1910. Price, $5.00.

This is a most timely and highly important book. It handles delicately, but explicitly, a vital subject which, through false notions, is touched if at all most gingerly by the text-book writers. Every doctor and teacher and mother should read it. I quote from the introductory chapter:

"* * * The sexual life of woman is considered both in relation to the female genital organs, and in relation to the feminine organism as a whole; in relation both to the physical and to the mental development of the individual; and in relation alike to the state of health and to the processes of disease. Thus from the standpoint of clinical investigation and of practical experience, the book will be a contribution towards the solution of the sexual problem, nowadays recognized as one of supreme importance."

